# Unilateral Corneal Ulcer in a 2-Month-Old Infant Without Identifiable Predisposing Factors: A Case Report

**DOI:** 10.3390/jcm15218420

**Published:** 2026-10-29

**Authors:** Dunja Bajtl, Vedran Nemet, Ivanka Maduna, Suzana Matić, Maja Vinković, Dubravka Biuk, Marija Jelić Vuković

**Affiliations:** 1 Department of Ophthalmology, Osijek University Hospital Centre, 31000 Osijek, Croatia; dunja.bajtl@kbco.hr (D.B.); suzimatic72@gmail.com (S.M.); majavinkovic77@gmail.com (M.V.); dubravka.biuk@gmail.com (D.B.); marija.jelic.vukovic@gmail.com (M.J.V.); 2 Faculty of Medicine, Josip Juraj Strossmayer University of Osijek, 31000 Osijek, Croatia; ivankamadun@gmail.com; 3 Health Centre of Osijek-Baranja County, 31000 Osijek, Croatia

**Keywords:** corneal ulcer, infant, pediatric keratitis, empirical antimicrobial therapy, infectious keratitis, corneal scarring

## Abstract

**Background/Objectives**: To describe the presentation, diagnostic evaluation, management, and clinical outcome of unilateral corneal ulcer in an otherwise healthy infant without identifiable predisposing factors. **Methods**: We report a case of a 2-month-old infant who presented with a visible white corneal lesion. Initial examination revealed a 2 × 1 mm fluorescein-positive corneal ulcer with a surrounding stromal infiltrate. There was mild conjunctival injection without ocular discharge. The anterior chamber was deep, without cells or flare. Corneal scraping was deferred because specimen collection would have required sedation or general anesthesia. Consequently, the microbial etiology remained unconfirmed, and empirical antimicrobial treatment was initiated. **Results**: The patient was treated with topical azithromycin, tobramycin, moxifloxacin, and aciclovir, as well as liposomal ozonized oil eye drops. During empirical treatment and close clinical monitoring, the corneal epithelial defect resolved within seven days, and the surrounding stromal infiltrate progressively decreased in size and opacity. At the one-month follow-up, a small residual stromal scar remained outside the visual axis. **Conclusions**: This case illustrates the diagnostic and therapeutic challenges of managing corneal ulcer in early infancy when microbiological confirmation is unavailable. The favorable clinical course observed during empirical treatment and close monitoring does not establish an infectious etiology or demonstrate a causal effect of antimicrobial therapy.

## 1. Introduction

Infectious keratitis is a potentially sight-threatening condition that may result in corneal ulcer, scarring, and permanent visual impairment. A recent systematic review and meta-analysis involving 58 studies and more than 134,000 patients reported that bacterial infections accounted for approximately 44% of infectious keratitis cases worldwide, although considerable geographical variation was observed [1]. However, these findings reflect the broader population and may not be directly applicable to neonates and infants. Pediatric infectious keratitis has been reported to account for approximately 13% of all infectious keratitis cases, although epidemiological data concerning infants and very young children remain limited [2]. In a retrospective study involving 57 children younger than two years, Soleimani et al. identified predisposing factors in 68.4% of cases, while corneal thinning and perforation occurred in 50.9% and 22.8% of cases, respectively [2]. The main predisposing factors include ocular trauma, previous ocular surgery, lagophthalmos, nasolacrimal duct obstruction, prematurity, and extended stay in a neonatal intensive care unit [2]. In this age group, infectious keratitis is most commonly bacterial in origin, followed by viral infections, whereas fungal and *Acanthamoeba* keratitis are less frequently reported [2].

Infectious keratitis in neonates and infants presents substantial diagnostic and therapeutic challenges because relatively few topical antimicrobial agents are approved for this age group. Furthermore, diagnostic procedures such as corneal scraping and in vivo confocal microscopy may be difficult to perform because of limited patient cooperation and may require sedation or general anesthesia.

Severe complications of infectious keratitis include corneal thinning, perforation, and endophthalmitis, some of which may require surgical intervention. Corneal healing may be accompanied by residual stromal scarring or neovascularization, potentially affecting visual development. Scarring involving or distorting the visual axis may contribute to amblyopia.

We report a case of unilateral corneal ulcer in a 2-month-old infant without identifiable predisposing factors, in whom progressive clinical improvement was observed during empirical antimicrobial treatment and close monitoring.

This case report was prepared in accordance with the CARE reporting guidelines. The completed CARE checklist is provided as Supplementary File S1.

## 2. Case Presentation

In April 2026, a 2-month-old female presented to the Osijek University Hospital Centre after her parents noticed a small white lesion on the cornea of her right eye (RE) earlier that day. The infant was delivered vaginally at 38 weeks of gestation, with a birth weight of 3100 g and Apgar scores of 10/10. Both parents and the patient’s sibling were reportedly healthy. Early neurodevelopment was unremarkable. The parents reported no history of head or ocular trauma, recent international travel, or exposure to pets, as well as no prior ocular surgeries or ocular surface diseases.

**Figure 1:**
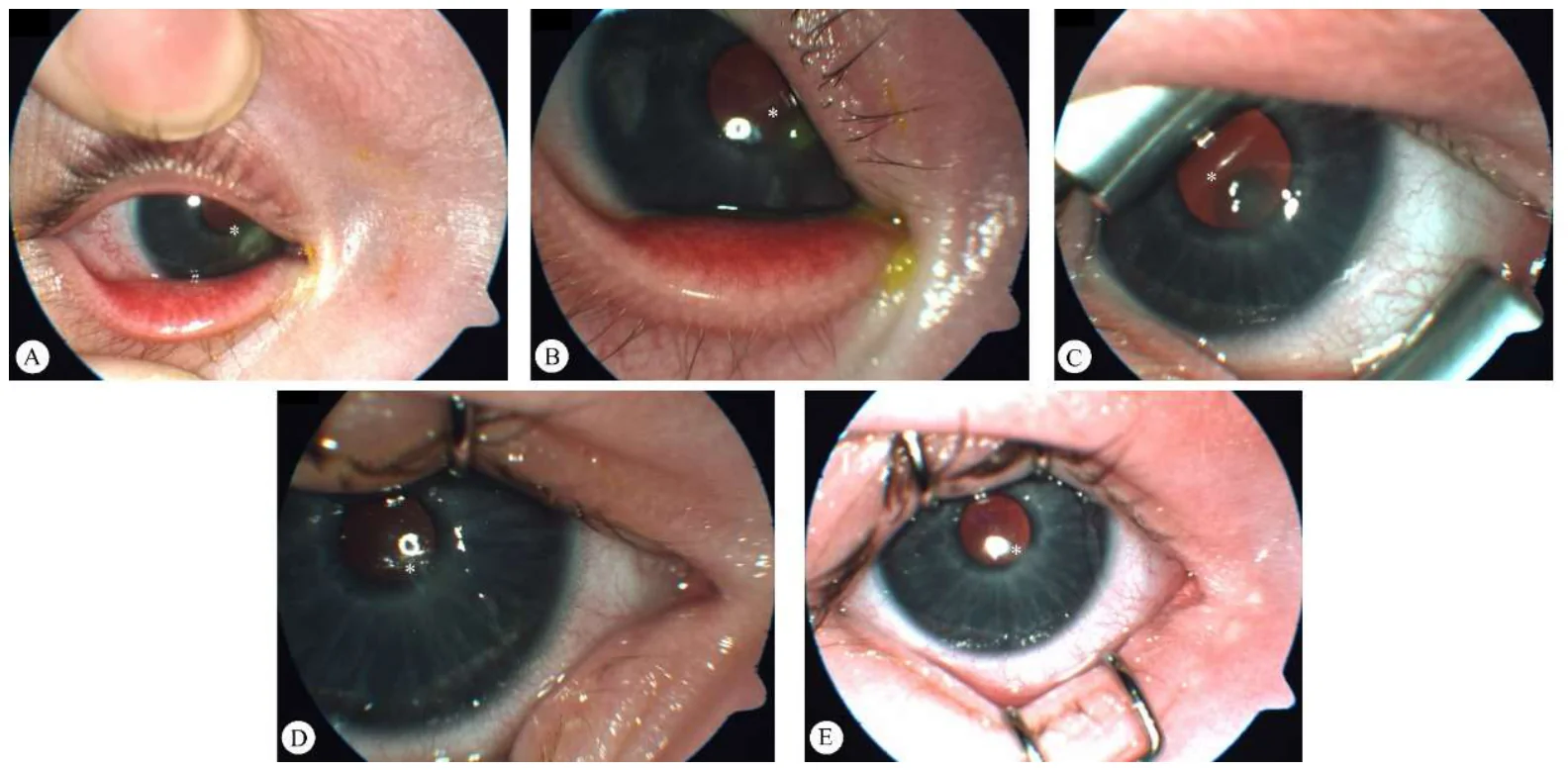
Serial anterior segment photographs of the right eye. (**A**) Initial presentation; (**B**) day 4; (**C**) day 7; (**D**) day 14; and (**E**) one month after presentation. White asterisks indicate the location of the initial corneal lesion or residual stromal opacity.

Slit-lamp examination of the RE revealed a 2 × 1 mm fluorescein-positive corneal ulcer surrounded by a stromal infiltrate (Figure 1A). All anterior segment photographs were captured using an FF 450plus fundus camera (Carl Zeiss Meditec AG, Jena, Germany). There was mild conjunctival injection, without ocular discharge. The anterior chamber was deep, without cells or flare. Examination of the iris, lens, and fundus of the right eye was unremarkable, as was anterior and posterior segment examination of the left eye.

Both upper and lower eyelids were everted to assess for retained foreign bodies and trichiasis, neither of which was identified. Corneal sensation was also assessed clinically; however, the infant’s young age and inability to cooperate prevented reliable interpretation of the blinking response to corneal stimulation. Eyelid position was normal bilaterally, with no evidence of lagophthalmos. There was no epiphora or evidence of nasolacrimal duct obstruction, and no periocular skin abnormalities were observed. The mother reported a history of recurrent herpes labialis; however, this information was based solely on maternal recollection and had not been laboratory-confirmed. Maternal CMV serological status was unknown because no previous maternal CMV testing had been performed. There was no reported recent contact with individuals with active herpes labialis and no history of birth trauma.

Conjunctival swabs were collected for bacterial and fungal culture, as well as *Chlamydia trachomatis* polymerase chain reaction (PCR), before initiation of topical antimicrobial therapy. Corneal scraping was not performed because it would have required sedation or general anesthesia. Instead, empirical broad-spectrum antimicrobial therapy was prescribed, along with frequent clinical follow-ups. Corneal scraping was to be reconsidered if the lesion deteriorated or failed to improve with empirical treatment.

The patient was hospitalized for seven days for treatment of corneal ulcer and close monitoring. Empirical topical treatment consisted of azithromycin (15 mg/g) twice daily for three days, tobramycin (3 mg/mL) five times daily for 14 days, moxifloxacin (5 mg/mL) three times daily for 14 days, and liposomal ozonized oil eye drops (Ozosept^®^, Proximum, Zagreb, Croatia) five times daily for 14 days. Because herpetic keratitis could not be excluded, topical aciclovir ointment (30 mg/g) was also administered five times daily for 14 days.

All conjunctival microbiological tests were negative. Laboratory investigations at admission revealed leukocytosis (20.6 × 10^9^/L; reference range: 6.0–16.0 × 10^9^/L) with an increased lymphocyte proportion (75.2%; reference range: 32.0–68.0%). CRP was within the reference range (0.6 mg/L; reference value < 2.8 mg/L). The infant was clinically well, afebrile, and showed no signs of systemic infection. On the final day of hospitalization, repeat laboratory testing showed a leukocyte count of 14.5 × 10^9^/L and a lymphocyte proportion of 65.0%, both within the laboratory reference intervals, while CRP remained unchanged at 0.6 mg/L. Blood culture was sterile. Serological testing was performed using a chemiluminescent immunoassay (CLIA). HSV-1/2 IgM (<0.5 COI; positive ≥ 1.1 COI), HSV-1 IgG (0.208 COI; positive ≥ 1.0 COI), and HSV-2 IgG (0.137 COI; positive ≥ 1.0 COI) were negative. CMV serology revealed positive IgG (3.56 IU/mL; positive ≥ 1.20 IU/mL) and negative IgM (0.184 AU/mL; positive ≥ 4.20 AU/mL). Negative infant HSV serology did not definitively exclude ocular HSV infection in the absence of corneal PCR testing.

The infant was examined by a pediatrician, who found no systemic abnormalities or clinical signs of disease apart from the previously described ocular findings. Feeding patterns remained normal, with regular formula intake and normal urine output.

Regular ophthalmological and pediatric examinations during the one-week admission, followed by daily outpatient ophthalmological and pediatric assessments for the remainder of the 14-day treatment period, showed significant improvement of the corneal findings (Figure 1B). Seven days after initial presentation, slit-lamp examination revealed minimal conjunctival injection, complete epithelialization of the corneal defect, and a reduction in the stromal infiltrate to 1 × 1 mm (Figure 1C). Fourteen days after presentation, conjunctival injection had resolved, the stromal infiltrate had further decreased in size and opacity, and the corneal epithelium remained fluorescein-negative, while the anterior chamber remained deep and quiet (Figure 1D). One month after the initial presentation, slit-lamp examination showed a quiet right eye, with a small, oval stromal scar located inferonasally to the pupil and outside the visual axis (Figure 1E).

**Figure 2:**
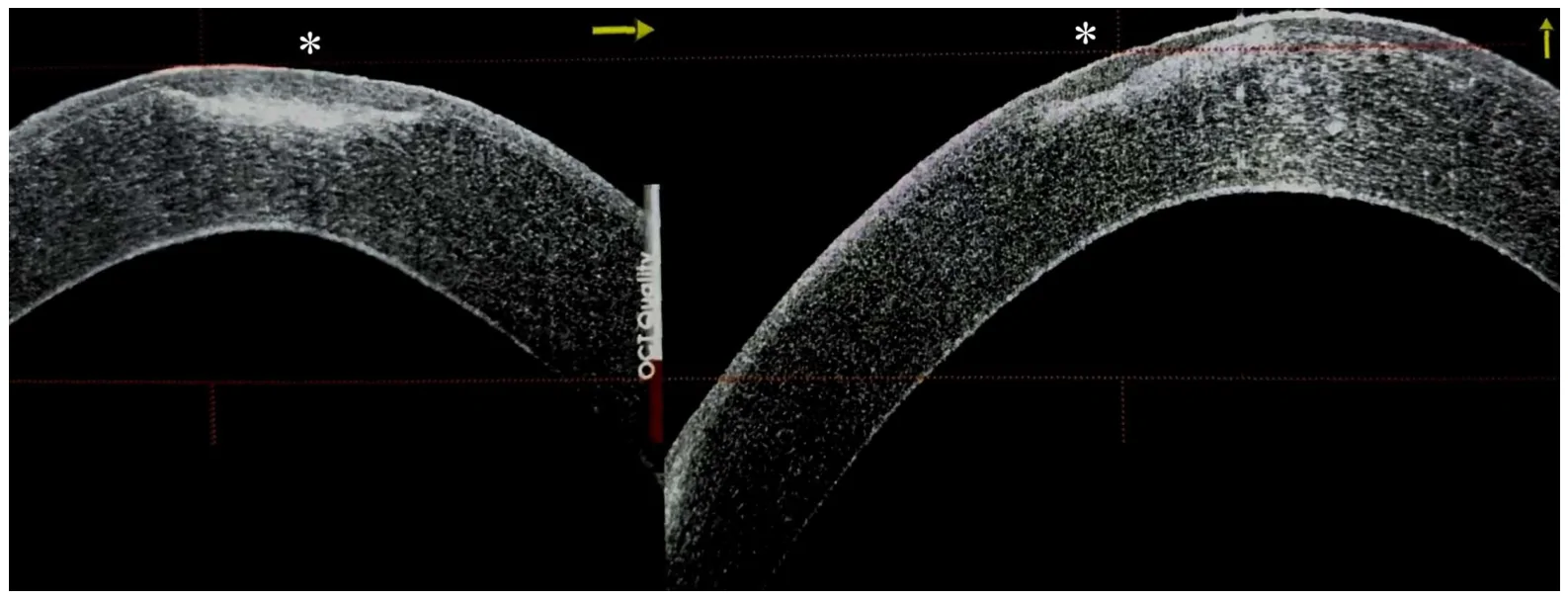
Anterior-segment optical coherence tomography of the right eye obtained 11 days after presentation, demonstrating focal anterior stromal hyperreflectivity with an intact overlying epithelium. White asterisks indicate the affected corneal region. Yellow arrowheads represent horizontal and vertical direction of the OCT cuts.

Anterior segment optical coherence tomography (AS-OCT) (Optovue Avanti XR, Optovue Inc., Fremont, CA, USA) performed 11 days after the initial presentation showed focal hyperreflectivity within the affected anterior corneal stroma, with an intact overlying epithelium. There was localized hyporeflectivity in the underlying stroma, with a well-defined demarcation between the affected and unaffected corneal tissue (Figure 2).

Additional follow-up examinations were performed in June and October 2026, approximately two and six months after the initial presentation, respectively. At both visits, the infant demonstrated age-appropriate visual tracking and fixation, without resistance to monocular occlusion of either eye. Ocular motility was unrestricted, and Hirschberg testing indicated orthotropia. Slit-lamp examination of the right eye revealed a fluorescein-negative cornea with a progressively fading inferonasal parapupillary stromal opacity, a deep and clear anterior chamber, and a transparent crystalline lens. At the October follow-up, when the infant was almost eight months old, corneal stimulation with a cotton tip elicited a blink response, making a profound corneal sensory deficit less likely. Cycloplegic retinoscopy at this follow-up revealed +0.50 D sphere/+1.50 D cylinder × 90° in the right eye and +1.00 D sphere/+1.50 D cylinder × 90° in the left eye. Although formal visual acuity testing was not feasible at this age, the clinical findings were reassuring, and continued follow-up is planned to monitor visual development and detect possible amblyopia.

The clinical course, diagnostic assessments, treatment, and follow-up findings are summarized in Table 1.

Tobramycin, moxifloxacin, and aciclovir ophthalmic ointment were administered off-label with respect to the patient’s age. Additionally, liposomal ozonized oil drops (Ozosept^®^, Proximum, Zagreb, Croatia) were administered despite the absence of established pediatric safety data, as the manufacturer indicates that their use in children has not been adequately investigated.

Treatment safety and tolerability were monitored through daily ophthalmological and pediatric examinations throughout the entire 14-day treatment period. No clinically apparent local adverse effects were observed during ophthalmological examinations, and no systemic adverse effects were identified during pediatric assessments.

## 3. Discussion

Corneal ulcer in infants younger than six months of age is rarely reported in the literature. Potential causes include neurotrophic keratopathy associated with congenital corneal hypoesthesia or herpetic infection, ocular trauma, congenital eyelid abnormalities (including entropion, eyelid coloboma, and tarsal kink syndrome), and exposure keratopathy [2].

Studies investigating neonatal and fetal wound healing have described age-dependent differences in cellular proliferation, inflammatory responses, and scar formation [3,4,5]. These observations may have implications for corneal epithelial repair, although direct evidence concerning corneal wound healing in human infants remains limited.

Although the lesion lacked typical dendritic morphology, HSV keratitis was considered because of the clinical presentation and the reported maternal history of herpes labialis. HSV-1 and HSV-2 infections can occur during the neonatal period. Primary ocular infection, present in the mucocutaneous form, is most commonly related to direct contact with mucous membranes. The infection is often subclinical, and may present with unilateral symptoms such as transient blepharitis, follicular conjunctivitis, and epithelial keratitis [6]. In our patient, anti-HSV-1/2 IgM and IgG antibodies were all negative; however, the absence of corneal sampling for HSV PCR prevented definitive microbiological exclusion.

Corneal scraping represents the main diagnostic procedure for infectious keratitis; however, it requires a level of patient cooperation. Soleimani et al. reported negative bacterial cultures in up to 60% of corneal specimens obtained from children aged two years or younger [2]. Culture positivity was associated with infiltrate ≥ 2 mm. In patients with herpetic keratitis, HSV PCR performed on corneal specimens has been reported to have high specificity [2]. Conjunctival swabs, although less invasive, are not a substitute for direct corneal sampling, as conjunctival cultures have demonstrated limited sensitivity and specificity in the diagnosis of keratitis [7,8]. Consequently, negative conjunctival cultures cannot exclude the presence of causative microorganisms within the corneal lesion, although they may occasionally yield supplementary microbiological information [9]. In our patient, conjunctival specimens were obtained as a minimally invasive supplementary investigation. Their negative results were interpreted cautiously because the absence of direct corneal sampling prevented microbiological confirmation or exclusion of an infectious etiology.

Although the lesion measured 2 × 1 mm and therefore reached the reported 2-mm threshold in one dimension, lesion size alone was not considered sufficient to determine the need for corneal scraping. In the present case, the decision to defer corneal sampling was based on an individualized assessment of the lesion’s peripheral location, mild conjunctival injection without ocular discharge, absence of anterior chamber inflammation, and availability of close clinical monitoring, weighed against the anticipated need for sedation or general anesthesia in a two-month-old infant. This decision should not be interpreted as a general recommendation to omit microbiological sampling in infants with similarly sized corneal lesions. The subsequent rapid clinical improvement without progression of the infiltrate or development of anterior chamber inflammation allowed continued conservative management without corneal sampling. In vivo confocal microscopy is not available at our centre.

The interpretation of viral serological findings in early infancy requires caution because maternally derived IgG antibodies may persist for several months after birth. Although the mother reported recurrent herpes labialis, this history was not supported by laboratory confirmation of HSV infection. Differences in maternal antibody concentrations, transplacental transfer, postnatal antibody decline, and assay characteristics may also influence the detection of HSV-specific IgG in early infancy. Similarly, positive CMV IgG in the presence of negative CMV IgM may reflect passively transferred maternal antibodies rather than active infection. Maternal CMV serological status remained unknown because no previous maternal serological testing had been performed. Importantly, these serological findings alone cannot definitively establish or exclude a viral etiology of the corneal lesion.

Given the patient’s very young age and the potentially rapid progression and vision-threatening consequences of microbial keratitis in infancy, broad empirical antimicrobial coverage was selected pending clinical response. Chaurasia et al. reported *Staphylococcus* spp., *Streptococcus* spp., and *Pseudomonas aeruginosa* as the most common causes of neonatal infectious keratitis [10]. In our case, azithromycin was included to provide empirical coverage for possible *Chlamydia trachomatis* infection. It was administered twice daily for three days in accordance with the preparation’s approved dosing schedule. Furthermore, azithromycin was the only topical ophthalmic antibiotic authorized for use in this patient’s age group in Croatia. Moxifloxacin was administered primarily to provide coverage against *Staphylococcus* spp. and *Streptococcus* spp. as potential causative microorganisms. Additionally, moxifloxacin has demonstrated greater ocular tissue penetration than several other topical fluoroquinolones [11]. Tobramycin was included to provide additional empirical coverage against Gram-negative pathogens, including *Pseudomonas aeruginosa.* The dosing frequencies of tobramycin (five times daily) and moxifloxacin (three times daily) were individualized primarily on the basis of the small, peripheral corneal lesion, limited inflammatory response, and absence of anterior chamber inflammation. The patient’s young age and the potential for cumulative ocular surface toxicity associated with multiple concurrent topical preparations were additional considerations. Although the concurrent administration of multiple topical preparations may increase the risk of ocular surface irritation or epithelial toxicity, no clinically apparent local adverse effects were identified during daily ophthalmological examinations. The simultaneous administration of three topical antibiotics represented an individualized empirical treatment decision, rather than a standard therapeutic regimen. Liposomal ozonized oil eye drops (Ozosept^®^, Proximum, Zagreb, Croatia) were administered as an adjunctive treatment. Previous preclinical and limited clinical studies have reported their antimicrobial and ocular-surface effects [12]. Scaramuzzi et al. compared safety and tolerability of liposomal ozonized oil with those of a topical antibiotic in preterm infants, and found no significant difference in adverse effects in this age group; however, those findings do not establish the efficacy or safety of this preparation for treating corneal ulcers in 2-month-old infants [13].

Regular ophthalmological follow-up is particularly important because residual corneal opacity may induce irregular astigmatism, higher-order aberrations, and visual deprivation, increasing the risk of amblyopia [2]. Follow-up examinations will focus on age-appropriate visual function, refractive changes, and signs of amblyopia, with treatment initiated if indicated.

An additional differential diagnosis in this case was a corneal epithelial defect, potentially resulting from unrecognized minor trauma, accompanied by a secondary sterile inflammatory infiltrate. Although no history of ocular trauma was reported, a noninfectious etiology could not be definitively excluded. Furthermore, because no causative microorganism was identified and multiple topical preparations were administered simultaneously, the observed clinical improvement cannot be attributed conclusively to antimicrobial treatment. Spontaneous epithelial healing may also have contributed to the favorable clinical course. Therefore, this report should be interpreted as an observational description of corneal ulcer resolution during empirical treatment and close clinical monitoring, rather than evidence of the efficacy of a particular antimicrobial regimen.

Although no predisposing factors were identified during the initial clinical evaluation, the assessment of corneal sensation was limited by the patient’s young age and inability to cooperate. Therefore, congenital corneal hypoesthesia or neurotrophic keratopathy could not be definitively excluded at presentation. Nevertheless, corneal stimulation with a cotton tip elicited a blink response at the subsequent follow-up examination at almost eight months of age, making a profound persistent corneal sensory deficit less likely, although subtle hypoesthesia could not be excluded by this qualitative assessment.

Additional limitations include the absence of direct corneal microbiological testing and the inability to determine the contribution of individual topical preparations to the observed clinical course. Although qualitative visual assessments and cycloplegic retinoscopy were reassuring at the six-month follow-up, formal visual acuity testing was not feasible because of the patient’s age, and longer-term visual development remains to be established.

## 4. Conclusions

This case illustrates the diagnostic and therapeutic challenges of managing corneal ulcer in early infancy, particularly when direct corneal sampling would require sedation or general anesthesia. Progressive epithelial healing and reduction of the residual stromal opacity were observed during empirical topical treatment and close clinical monitoring. At the six-month follow-up, qualitative visual assessments and cycloplegic refractive findings were reassuring. However, the absence of microbiological confirmation prevents definitive attribution of the lesion to an infectious etiology or of the favorable clinical course to antimicrobial treatment. A noninfectious epithelial defect with secondary sterile inflammation remains a possible alternative explanation. Continued ophthalmological follow-up is necessary to monitor visual development and detect potential amblyopia.

## Figures and Tables

**Table 1:** Timeline of clinical presentation, treatment, and follow-up. Follow-up intervals are approximate where expressed in months.

Time Point	Clinical Findings and Investigations	Treatment and Outcome
Initial presentation (Day 0)	A 2 × 1 mm fluorescein-positive corneal ulcer with a surrounding stromal infiltrate. Mild conjunctival injection; anterior chamber deep and quiet. Microbiological specimens collected and laboratory investigations performed.	Hospitalization and initiation of empirical topical antimicrobial and antiviral therapy.
Day 4	Clinical reassessment and photographic documentation (Figure 1B).	Continued treatment and close monitoring.
Day 7	Complete epithelialization; residual infiltrate measuring 1 × 1 mm; minimal conjunctival injection (Figure 1C).	Significant clinical improvement.
Day 11	AS-OCT demonstrated focal anterior stromal hyperreflectivity with intact overlying epithelium (Figure 2).	Persistent stromal changes without epithelial defect.
Day 14	Further reduction in stromal infiltrate size and opacity; negative fluorescein staining (Figure 1D).	Continued improvement.
1 month	Small inferonasal stromal opacity outside the visual axis (Figure 1E).	Persistent epithelial integrity with a small residual stromal opacity.
2 months	Appropriate visual tracking, no resistance to monocular occlusion, orthotropia, and no apparent ocular motility restriction. Fluorescein-negative cornea with less opaque residual stromal lesion.	Continued improvement and reassuring qualitative visual behavior.
6 months	Similar visual behavior and ocular alignment. Further reduction in stromal opacity. Normal left anterior segment and bilateral fundus examination. Cycloplegic retinoscopy: RE +0.50/+1.50 × 90°; LE +1.00/+1.50 × 90°.	Sustained epithelial integrity, further reduction in residual stromal opacity, and reassuring clinical findings.

## Data Availability

All data supporting the findings of this case report are included in the manuscript.

## Supplementary Materials

The following supporting information can be downloaded at: https://www.mdpi.com/article/10.3390/jcm15218420/s1, The completed CARE (CAse REport) checklist is provided as Supplementary File S1.

## Abbreviations

The following abbreviations are used in this manuscript:

AS-OCT	Anterior-segment optical coherence tomography
CLIA	Chemiluminescent immunoassay
CMV	Cytomegalovirus
CRP	C-reactive protein
HSV	Herpes simplex virus
IgG	Immunoglobulin G
IgM	Immunoglobulin M
LE	Left eye
PCR	Polymerase chain reaction
RE	Right eye
