# Supplementary material for: Unilateral Corneal Ulcer in a 2-Month-Old Infant Without Identifiable Predisposing Factors: A Case Report
Source: J Clin Med. 2026 Oct 29;15(21):8420. doi: 10.3390/jcm15218420 (PMC13764282; doi:10.3390/jcm15218420)
Supplement: Supplementary file 1 [file jcm-15-08420-s001.zip › jcm-4634081-supplementary.pdf]

CARE case report guidelines

CARE Checklist of information to include when writing a case report

(cc) BY-NC-ND

Topic	Item	Checklist item description	Reported on Line
Title	1	The diagnosis or intervention of primary focus followed by the words "case report"	2-3
Key Words	2	2 to 5 key words that identify diagnoses or interventions in this case report, including "case report"	34-35
Abstract (no references)	3a	Introduction: What is unique about this case and what does it add to the scientific literature?	16-18
	3b	Main symptoms and/or important clinical findings	18-24
	3c	The main diagnoses, therapeutic interventions, and outcomes	24-29
	3d	Conclusion—What is the main "take-away" lesson(s) from this case?	29-33
Introduction	4	One or two paragraphs summarizing why this case is unique (**may include references**)	38-67
Patient Information	5a	De-identified patient specific information.	69
	5b	Primary concerns and symptoms of the patient.	70
	5c	Medical, family, and psycho-social history including relevant genetic information	71-75
	5d	Relevant past interventions with outcomes	73-75
Clinical Findings	6	Describe significant physical examination (PE) and important clinical findings.	76-81
Timeline	7	Historical and current information from this episode of care organized as a timeline	162-163
Diagnostic Assessment	8a	Diagnostic testing (such as PE, laboratory testing, imaging, surveys).	97-102; 109-121
	8b	Diagnostic challenges (such as access to testing, financial, or cultural)	99-102
	8c	Diagnosis (including other diagnoses considered)	103-108
	8d	Prognosis (such as staging in oncology) where applicable	N/A
Therapeutic Intervention	9a	Types of therapeutic intervention (such as pharmacologic, surgical, preventive, self-care)	103-109
	9b	Administration of therapeutic intervention (such as dosage, strength, duration)	103-109
	9c	Changes in therapeutic intervention (with rationale)	N/A
Follow-up and Outcomes	10a	Clinician and patient-assessed outcomes (if available)	126-236
	10b	Important follow-up diagnostic and other test results	137-159
	10c	Intervention adherence and tolerability (How was this assessed?)	172-175
	10d	Adverse and unanticipated events	172-175
Discussion	11a	A scientific discussion of the strengths AND limitations associated with this case report	177-289
	11b	Discussion of the relevant medical literature **with references**.	177-289
	11c	The scientific rationale for any conclusions (including assessment of possible causes)	262-283
	11d	The primary "take-away" lessons of this case report (without references) in a one paragraph conclusion	291-300
Patient Perspective	12	The patient should share their perspective in one to two paragraphs on the treatment(s) they received	N/A
Informed Consent	13	Did the patient give informed consent? Please provide if requested	Yes ■ No ☐
